# Selection and Adaptation to Urban Food Resources Promote Spotted Dove (*Streptopelia chinensis*) as a Common Species in Urban Habitats

**DOI:** 10.1002/ece3.71773

**Published:** 2025-07-10

**Authors:** Mengjie Lu, Xueli Zhang, Jiahan Ruan, Wanyou Li, Mingwang Chen, Long Ma, Zhen Zhang, Bicai Guan, Luzhang Ruan

**Affiliations:** ^1^ School of Life Sciences Nanchang China; ^2^ Guangdong Maoming Health Vocational College Maoming China; ^3^ State Ministry of Education Key Laboratory of Poyang Lake Environment and Resource Utilization Nanchang University Nanchang China

**Keywords:** dietary composition, DNA metabarcoding, seasonal dietary variation, selective foraging, urbanization

## Abstract

Rapid urban development has almost completely reshaped the original landscape. Then birds are required to adapt to urban food resources for their survival in the city. In recent years, spotted doves have become increasingly common in urban habitats, but their dietary composition and variation are unclear. The DNA metabarcoding technique was applied to identify the primary food components of spotted doves ( *Streptopelia chinensis* ) in Nanchang, China. A total of 100 plant species were identified from 251 spotted dove fecal samples throughout four seasons (2020–2021). Spotted doves foraged *Cinnamomum camphora* seeds assigned to the Lauraceae family in the highest proportion throughout the year, suggesting that urban spotted doves mainly depend on artificial greening plants as their main food source. Furthermore, the dietary composition of spotted doves varied with increasing or decreasing plant resources. In spring, spotted doves primarily consumed Gramineae plants when they were growing and seed‐bearing, while foraging the seeds of *Kummerowia striata* first in autumn. Although *Cinnamomum camphora* seeds were available in a year, which provided the conserved food, spotted doves would first choose to forage on fresh and high‐quality plants when food resources were adequate and stable. Our study confirmed that spotted doves can make use of urban food resources, but we still recommend maintaining the diversity of plants when greening to provide more food choices for urban birds. That will promote the survival of birds in urban habitats, resulting in a city where humans and birds coexist harmoniously.

## Graphical abstract

The manuscript described the dietary composition and variation of a common species—spotted dove in urban habitats, and discussed the constraint of urban food resources for it and provided recommendations for urban greening.

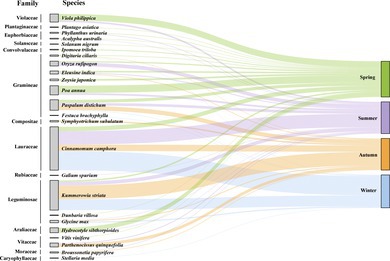

## 1 Introduction

Urbanization is proceeding at a rapid pace across the globe, with a tremendous impact on landscape features and the distribution of urban wildlife food resources (Fang et al. 2021; Kozlovsky et al. 2021). There are almost all artificial grasses and trees in urban environments, and only a few fauna can adapt as well as survive and reproduce successfully (Cooper et al. 2022). Studies about the ecology and adaptation of urban fauna often concentrate on birds because they are more prevalent in cities (Chamberlain et al. 2009; Pollock et al. 2017; Seress et al. 2018). Of the approximately 10,000 species of birds identified worldwide, 20% can survive successfully in urban habitats (Aronson et al. 2014). With the expansion of the urban range, more and more birds have to shift from nature to urban, such as the Chinese blackbird (*Turdus mandarinus*), light‐vented bulbul ( *Pycnonotus sinensis* ) and gray‐capped greenfinch (*gray‐capped greenfinch*) (Han et al. 2019; Xie et al. 2016). Most studies have analysed the effects of urban green space size (Matthies et al. 2017; Nielsen et al. 2014) and vegetation structure (Chang et al. 2017) on birds. However, few studies have analysed whether birds can adapt to food resources in urban habitats.

In urban habitats, acquiring nesting and food resources in the urban landscape may be a challenge for birds, especially for herbivorous birds (Ikin et al. 2012; Sander and McCurdy 2021; Vincze et al. 2019). Although some artificial green spaces (i.e., parks, gardens, university campuses) contribute to the survival of wildlife habitats (Pinho et al. 2021), these places are subject to greater anthropogenic disturbance and remain relatively homogeneous in terms of food resources. Food resources will directly affect the survival of birds in cities (Marinero et al. 2020). It is important to explore the dietary composition of birds to relieve their survival dilemma, which can better facilitate their adaptation to urban habitats (Liu et al. 2019; Mackenzie et al. 2014). Therefore, understanding the foraging items of birds can better help us plan urban greening, increasing the biodiversity of the city, and building a nature‐friendly city where humans and birds live in harmony (Hadi et al. 2024).

In order to adapt to urban habitats, birds may adjust their previous diets (in natural habitats) and search for alternative foods in urban habitats (Jarrett et al. 2020), which may lead to changes in the dietary preference of birds. Also, even in cities, the abundance and phenological period of various plants vary, leading to seasonal environments that have peaks of most Gramineae plants in spring and summer, seed plants that produce the maximum amount of food in autumn, while dropping below the levels characteristic of constant environments in winter (McNamara et al. 2008). As a result, dietary composition and diversity of herbivorous birds can typically shift seasonally in response to changes in plant availability (Thomas et al. 2020; Amponsah‐Mensah et al. 2019). Because optimal foraging theory suggests that animals must obtain a sufficient amount of high‐quality food as quickly and safely as possible when foraging (Charnov 1976; MacArthur and Pianka 1966). For example, some birds will forage on preferred plants (often with high nutritional content) when food is abundant and stable, while acquiring energy and nutrients by decreasing their selective preference when food is scarce (Hasui et al. 2009).

For exploring the dietary composition of urban birds, the DNA metabarcoding method based on high‐throughput sequencing has been widely mentioned (Bourbour et al. 2021; Garfinkel et al. 2022). Traditional methods such as gastrointestinal dissection or manual monitoring data are relatively incapable of rapidly and accurately characterizing the complex diet composition of birds (Yoshikawa and Osada 2015). In addition, it is unsustainable to access the dietary composition of birds using traditional destructive methods (Tang et al. 2022). With the rapid development of molecular biology techniques, DNA metabarcoding has emerged as one of the most popular and fastest‐growing methods of dietary studies due to its non‐lethal feature in recent years (Song and Proctor 2020). DNA metabarcoding can process a greater number of samples and identify multiple taxa in a shorter time (Drake et al. 2023; Kuang et al. 2023). As a result, DNA metabarcoding has been widely used for the dietary study of generalist birds (Schumm et al. 2023).

In the past, spotted doves ( *Streptopelia chinensis* , Scopoli, 1786) were commonly seen in rural areas (Pang 1980). Its primary food source is granular plant seeds such as rice grain, and spotted doves usually peck on the ground (Pang 1980; Yan and Ma 1992). Based on our observations over the years, the spotted dove is becoming a successful urban sedentary species with increasing numbers in the city (Han et al. 2019; Alan and Beth 2016). It requires further exploration as to what plants the spotted doves primarily take in urban environments. In addition, the spotted dove is characterized by its ability to breed throughout the year and its need to obtain adequate food resources and energy sources in all seasons (Sheng et al. 2024). This means it can directly reflect the effects of urban resources on bird diets in different seasons. The focus of this research was to explore the dietary composition and variation of spotted doves in urban habitats and to analyze constraints of urban food resources for them. Here, we propose the hypothesis: (1) spotted doves rely primarily on artificial greening plants for their main diet in the study area; (2) seasonal resource imbalances can influence the selection of spotted doves' foraging. Their preferred plants change in response to the availability of resources.

## 2 Materials and Methods

### 2.1 Study Site

**FIGURE 1:**
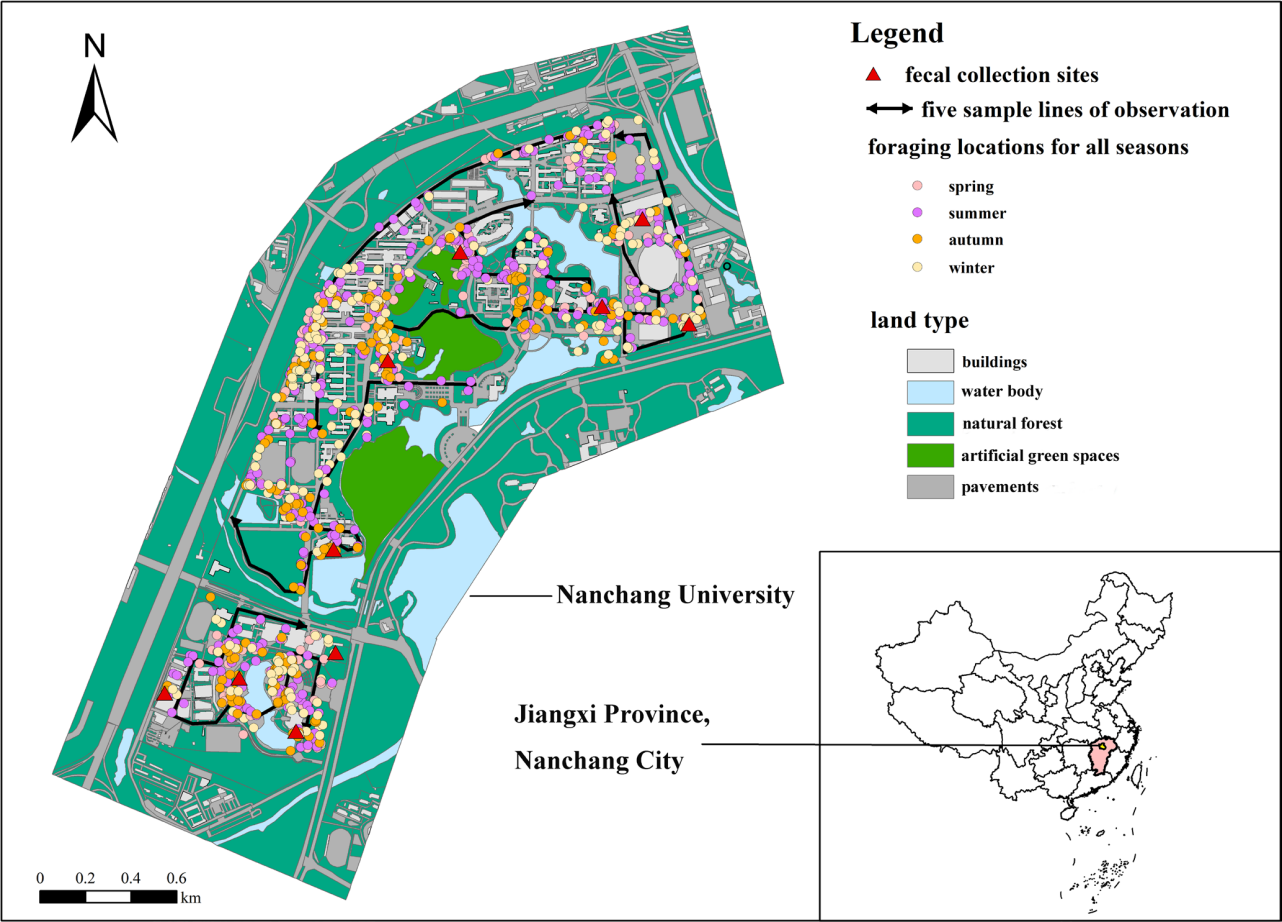
Fecal collection sites of the Nanchang University, Jiangxi Province, China. The vegetation sampling of four seasons were implemented from the locations of the foraging sites in the map.

The study was conducted in Qianhu (115°47′12″–115°38′34″ E, 28°39′4″–28°40′16″ N), located to the west of Nanchang City, Jiangxi Province, China (Figure 1). The climate is subtropical humid monsoon with evergreen vegetation in all seasons. The landscape is based on a plain; a large number of modern buildings and urban greening are concentrated here. The habitat can be classified into five main types, that is, buildings, water body, artificial green spaces, natural forests, and pavements. Among several habitat types, the frequency of application of plants in artificial green spaces is much higher than that of other land types due to their suitability as a haven for birds to reproduce and inhabit (Han et al. 2019). In the study area, the roadside is full of artificially planted *Cinnamomum camphora* . Because it is the city tree of Nanchang and is also the symbol of Nanchang University, *C. camphora* can be found everywhere.

### 2.2 Vegetation Sampling

Vegetation sampling was conducted in 2020–2021. A total of five walking sample lines were set up in the study area, all of which were within the range of 2–2.5 km in length. These sample lines basically covered all areas, and the survey dates included the early, middle, and late of each month. Sample line surveys were conducted from 6:00 to 9:00 a.m. and 17:00 to 19:00 p.m., and we walked along the sample lines at a speed of 1–2 km/h. When spotted doves were found foraging for more than 10 s at a site, we would record the number of pecking in a minute, and the latitude and longitude coordinates (GPS points) of the foraging site were also obtained.

In the foraging sites, we used 1 m × 1 m quadrats to sample vegetation composition in four seasons of 2020–2021. These vegetation quadrats were also set in early, middle, and late of each month. Each quadrat was separate. Then we recorded and identified plants at the lowest taxonomic level possible in quadrats. The relative coverage of plants within each quadrat was measured; it is the proportion of the coverage of a particular plant to the overall coverage of all plants. Relative coverage of herbaceous plants (%): coverage of a particular plant within the quadrat/total coverage of all plants within the quadrat × 100%. We did not record any plant species above 1.0 m high (excluded any trees or shrubs in each vegetation quadrat) because spotted doves usually forage on the ground from what we have observed, but we calculated the relative density of *C. camphora* (Nees ex Wall, 1831) seeds. Relative density of seeds of trees or shrubs (per/m^2^): the number of seeds of a particular plant in the quadrat/the area of the quadrat. We also used Schoener's niche overlap index (Schoener 1974) implemented in the “spaa” package in R software (Zhang 2016) with relative coverage of plants to compare the similarity of environmental plants in four seasons. In order to indicate the degree of urbanization in which each quadrat is situated, we also used ArcGIS (version 10.6) to calculate the Index of the Building and Pavement Proportion (0–20 m, %) of each quadrat. It is the proportion of impervious surfaces (including roads and buildings) within a 20 m radius of each foraging quadrat. To evaluate the correlation between relative density of *C. camphora* seeds and the Index of the Building and Pavement Proportion, a Pearson's rank correlation test was run.

### 2.3 Fecal Sample Collection

Fecal samples were collected, respectively, from March 2020 to February 2021. According to the foraging sites recorded during the sample‐line survey, we selected 10 fecal collection sites in the study area, which were evenly distributed as much as possible to ensure the integrity of sampling. The feces were collected from 6:00 to 9:00 a.m.; it was conducted two times a month, and we used binoculars to determine the foraging location of spotted doves. After the birds flew away, we would quickly collect fresh and superficial feces using a disposable plastic spoon to avoid contamination, and put them in tubes and labeled. The samples were immediately stored in an incubator with ice bags for transportation and then returned to store at −20°C with anhydrous ethanol in the laboratory.

### 2.4 DNA Extraction, PCR Amplifcation, Library Construction and Sequencing

Total DNA was extracted from feces samples using CTAB methods (Hou 2019). Universal primers were selected for the trnl of the plant chloroplast; the pair primers c (5′‐CGAATCGGTAGACGCTACG‐3′) and h (5′‐CCATTGAGTCTCTGCACCTATC‐3′) were suitable molecular markers for plant species identification (Taberlet et al. 2007). PCR amplification was performed in a total of 25 μL volume containing 1 μL DNA template, 2 μL of both forward and reverse primers (1.0 μL each), 13.5 μL of 2× Taq PCR MasterMix, and 9.5 μL water. The PCR procedure was as follows: 94°C for 5 min, followed by 35 cycles of denaturation at 94°C for 30 s, annealing at 58.5°C for 45 s, extension at 72°C for 1 min, and a final extension step at 72°C for 5 min. Three independent polymerase chain reactions (PCR) were performed for each sample, and 1% agarose gel electrophoresis was added to verify the amplification results. The resulting PCR amplifications were purified and sequenced by Personalbio Biotechnology Co. Ltd. (Shanghai, China) on an Illumina NovaSeq 6000 platform.

### 2.5 Bioinformatic Processing and Statistical Analyses

To identify taxonomic information of plant sequences from fecal samples, we established a DNA library of plants at Nanchang University. We obtained 118 plant sequences at the species level; 74 sequences of plant‐matching were queried from the GenBank database. For records not found by GenBank, 44 sequences of 44 plants were obtained by collecting and extracting DNA from plant samples, through PCR amplification and the generation sequencing by Shanghai Personalbio Biotechnology Co. Ltd.

Raw sequences were processed using QIIME 1.7.0 (Caporaso et al. 2010) for sequence quality filtering and removing the sequences with length < 100 bp. We clustered sequences at 97% similarity into Operational Taxonomic Units (OTUs) following the standard settings in USEARCH 7.0 (Edgar 2013). The sequences were annotated based on BLAST 2.2.7.0 (Camacho et al. 2009) in the DNA library to obtain classification information at various levels such as the family, genus, and species levels to identify the dietary composition of spotted doves. Moreover, when one sequence was associated with more than one taxon, it was assigned to the higher taxonomic level (genus or family).

Rarefaction curve could be checked to ensure the sufficient sequence sample depth in subsequent analyses (Chao et al. 2014), and we used Origin 2022 to calculate rarefaction curves for fecal data of all seasons.

The relative abundance was used to estimate the dietary composition of the animals, indicating the share and importance of a particular food OTUs in the diet, and it is the percentages of sequences assigned to a given OTUs for all samples (Kartzinel and Pringle 2015; Tang et al. 2022). Based on relative abundance, we analyzed the plant composition and diversity in feces. Dietary diversity indices (i.e., Shannon, Simpson, Pielou, and Levins) were performed using the “vegan” and “picante” packages of R software. The Bonferroni test was used to assess the difference in alpha diversity index between different seasons. *p* ≤ 0.05 indicated statistical significance. The bar charts were created using OriginPro software. In order to test whether the spotted dove is selective for environmental plants, we analyzed the relationship between pecking frequency and the relative coverage of the five plants (the top five species of relative abundance in feces, excluding plants not present in vegetation quadrat) in the quadrat. The Pearson correlation analysis was used. Taxa summary bar charts and Sankey plots exhibited the relative abundance of diet composition across months or seasons. Taxa summary bar charts, Sankey plots, boxplots, and Pearson correlation charts were generated using OriginPro.

## 3 Results

### 3.1 Sequencing Results and Dietary Composition

All 251 fecal samples were successfully sequenced using the Illumina platform, including 60 spring samples, 65 summer samples, 64 autumn samples, and 62 winter samples (Table 1). The libraries generated 2,726,663 raw sequences based on trnl amplification. After quality filtering, a total of 237 OTUs were obtained for subsequent analyses. Rarefaction curves tended to extend gently, indicating that our sample size of fecal samples and sequencing depth were effective at documenting the diverse diet (Figure S1).

**TABLE 1:** Fecal collection information of spotted doves in 2020–2021.

Season	Spring			Summer			Autumn			Winter		
Month	Mar	Apr	May	Jun	Jul	Aug	Sep	Oct	Nov	Dec	Jan	Feb
Quantity	16	23	21	19	23	23	19	21	24	19	20	23
Total	60			65			64			62		

**FIGURE 2:**
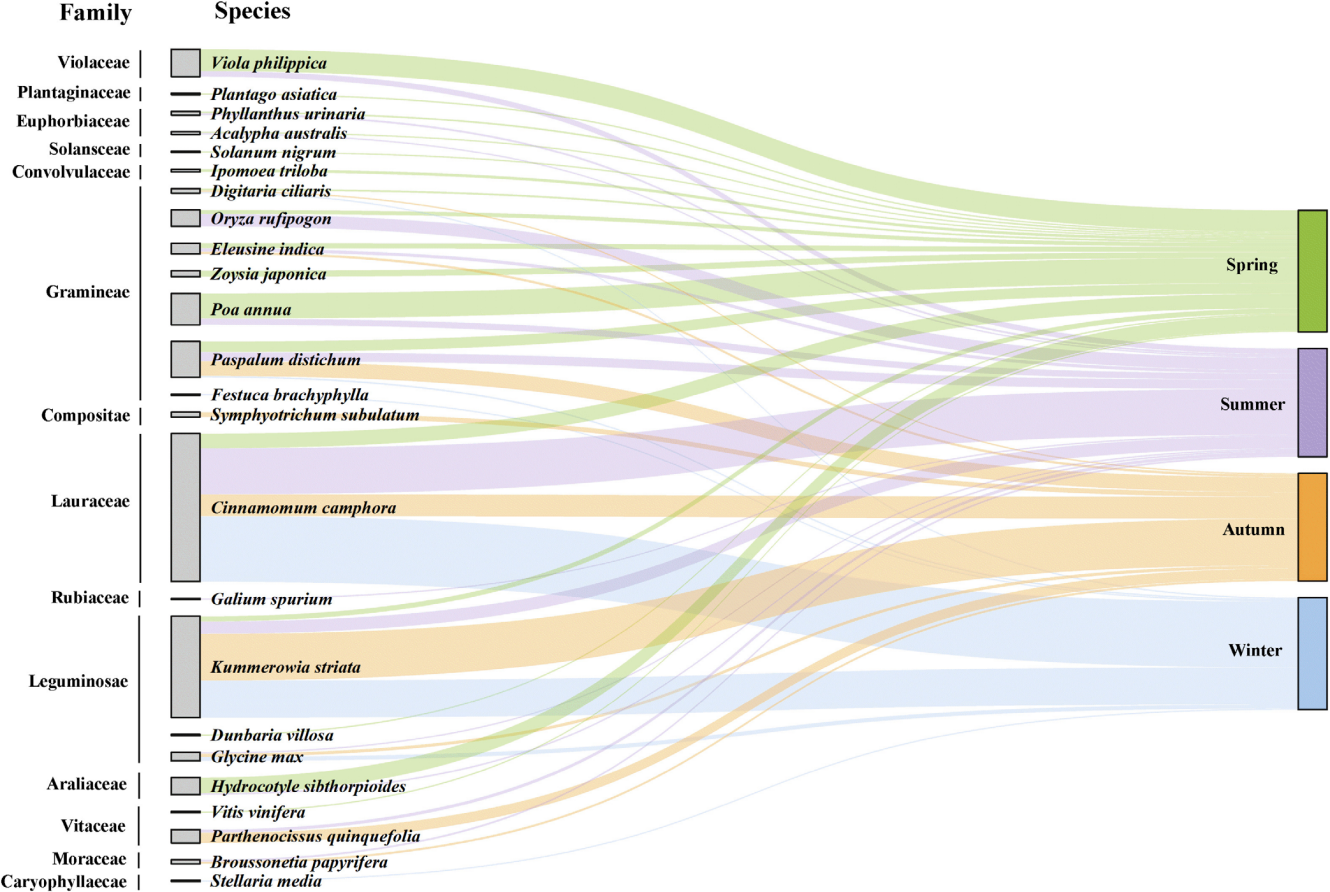
Sankey diagram showing associations between different seasons and plant foods at the level of family and species. The connecting bar width represents the relative abundance of dietary items among periods and dietary items.

The plants food items of spotted doves mainly originated from 98 species and 2 unidentified plants (*Pugionium* and *Chusquea*) that we put into genus classification. Families including Lauraceae, Leguminosae, and Gramineae were frequently found and contributed to the majority of plant food throughout the year, especially Lauraceae (Figure 2). The highest relative abundance was *Poa annua* (20.73%) in spring, followed by *Viola philippica* (18.15%) and *Hydrocotyle sibthorpioides* (12.93%). In summer, the top 4 plant items were *C. camphora* (38.06%), *Kummerowia striata* (10.18%), *Oryza rufipogon* (10.41%) and *Paspalum distichum* (7.53%). In addition, *C. camphora* dominated the diet of winter (54.32%) and was also easily detected in spring (12.10%) and autumn (18.00%). *K. striata* was observed at high relative abundance in autumn (38.69%) and winter (30.67%), but contributed the lowest in spring (4.22%) (Figure 2, Table 2).

**TABLE 2:** The relative abundance in the top 20 plant food items in the diet of the spotted dove. List of numbers in bold denote relative abundance > 5%.

Family	Species	Spring	Summer	Autumn	Winter
Lauraceae	*Cinnamomum camphora*	**12.10%**	**38.06%**	**18.00%**	**54.32%**
Leguminosae	*Kummerowia striata*	4.22%	**10.18%**	**38.69%**	**30.67%**
Gramineae	*Paspalum distichum*	**8.80%**	**7.53%**	**12.16%**	1.29%
Gramineae	*Poa annua*	**20.73%**	**5.26%**	0.26%	0.50%
Violaceae	*Viola philippica*	**18.15%**	4.66%	0.21%	0.31%
Gramineae	*Oryza rufipogon*	3.31%	**10.41%**	0.67%	0.70%
Araliaceae	*Hydrocotyle sibthorpioides*	**12.93%**	1.36%	0.56%	0.21%
Vitaceae	*Parthenocissus quinquefolia*	0.59%	2.62%	**8.53%**	0.04%
Gramineae	*Eleusine indica*	4.25%	2.66%	2.03%	0.61%
Leguminosae	*Glycine max*	0.15%	1.18%	2.54%	3.37%
Gramineae	*Digitaria ciliaris*	1.55%	0.59%	1.20%	1.11%
Euphorbiaceae	*Phyllanthus urinaria*	1.44%	1.63%	0.61%	0.34%
Compositae	*Symphyotrichum subulatum*	0%	0%	4.03%	0%
Moraceae	*Broussonetia papyrifera*	0.21%	1.61%	1.81%	0.04%
Convolvulaceae	*Ipomoea triloba*	2.34%	0.41%	0.21%	0.15%
Euphorbiaceae	*Acalypha australis*	1.10%	1.13%	0.53%	0%
Solanaceae	*Solanum nigrum*	1.12%	0.67%	0.07%	0.74%
Vitaceae	*Vitis vinifera*	1.14%	0.72%	0.05%	0.04%
Labiatae	*Pugionium cornutum*	0.11%	0.93%	0.19%	0.38%
Rubiaceae	*Galium spurium*	0.18%	1.15%	0.06%	0.07%

### 3.2 Temporal Variation in Diet

**FIGURE 3:**
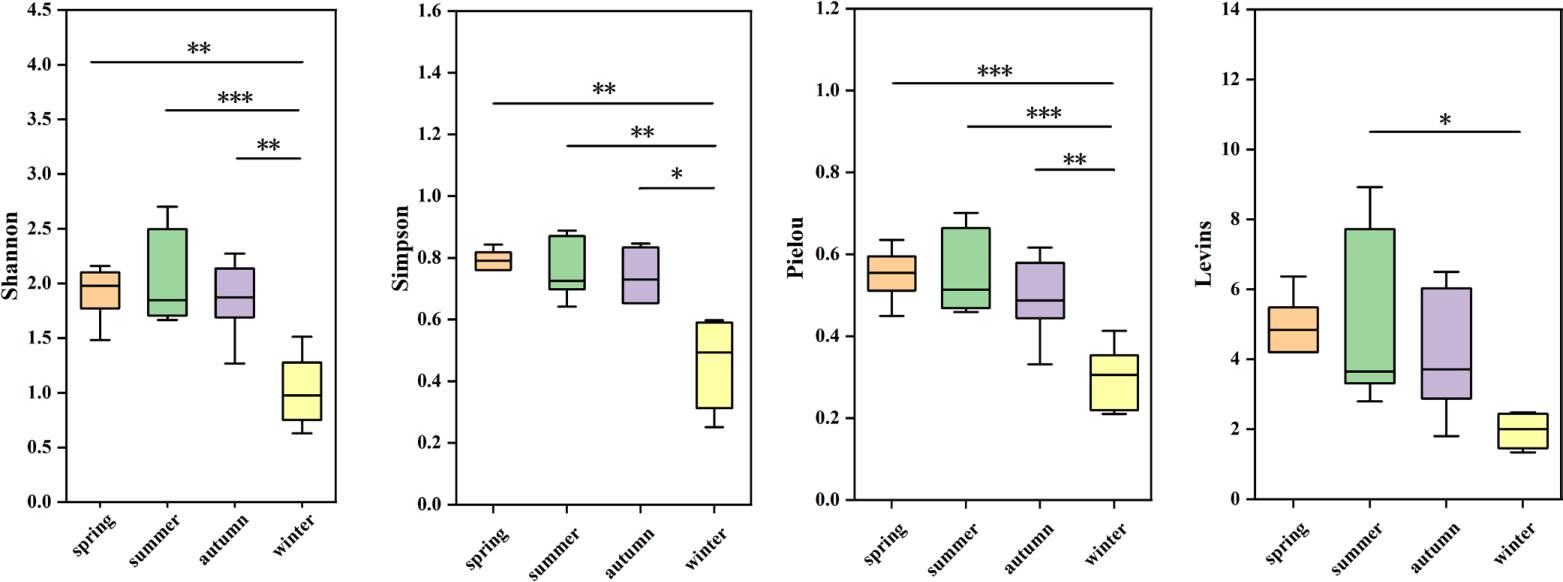
Diversity of plant foods in four seasons. Box‐and‐whisker plots for diversity in plant food item estimators (Shannon, Simpson, Pielou, Lewins indices). * indicates *p* < 0.05, ** indicates *p* < 0.01, ***indicates *p* < 0.001.

Overall, diversity indices (including Shannon, Simpson, Pielou, and Lewins) showed that intergroup differences in dietary diversity occurred among four seasons (Figure 3). The lowest diversity index was detected in winter. There were significant differences in the Shannon, Simpson, and Pielou diversity indices between winter and other seasons (*p* < 0.05) (Figure 3).

**FIGURE 4:**
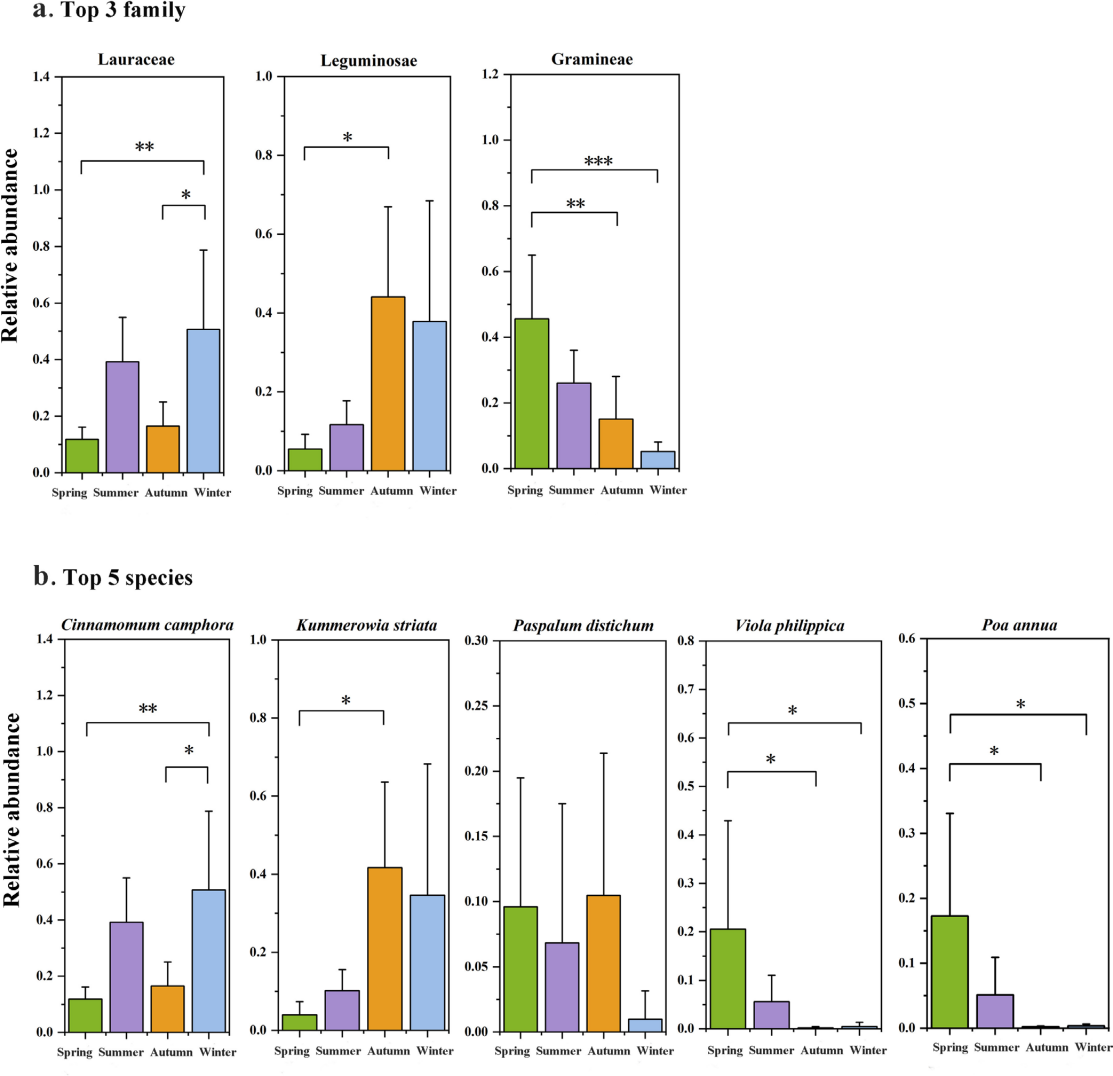
(a) Relative abundance of dominant plant foods (the top 3 familys are shown) of the fecal samples in four seasons. (b) Relative abundance of dominant plant foods (the top 5 speices are shown) of the fecal samples in four seasons. The error bars represent SD. * indicates *p* < 0.05, ** indicates *p* < 0.01, ***indicates *p* < 0.001.

As shown in Figure S2, we found the dietary composition that shifted a lot from spring to winter. Most notably, a lot of *C. camphora* in fecal samples was observed in June and July, and the proportion of *K. striata* increased from October. The fecal samples had *C. camphora* assigned to the Lauraceae family at a higher proportion in summer (38.06%) and winter (54.32%) than in spring (12.10%) and autumn (18.00%) (Figure 4a, Table 2). Leguminosae (mostly *K. striata* ) were mostly found in autumn and winter, and there was a significant difference in the relative abundance of Leguminosae between spring and autumn (*p* < 0.05) (Figure 4a,b). In spring, a diverse assemblage of Gramineae was commonly observed in fecal samples from spotted doves (Figure 4a). Among them, *P. annua* and *P. distichum* were consumed by spotted doves in the highest proportion. Furthermore, *V. philippica* and *P. annua* had higher relative abundance in spring (18.15%, 20.73%) than in autumn (0.21%; 0.26%) and winter samples (0.31%, 0.50%) (*p* < 0.05) (Figure 4b).

### 3.3 Correlation Between Vegetation Quadrats and Fecal Samples

**FIGURE 5:**
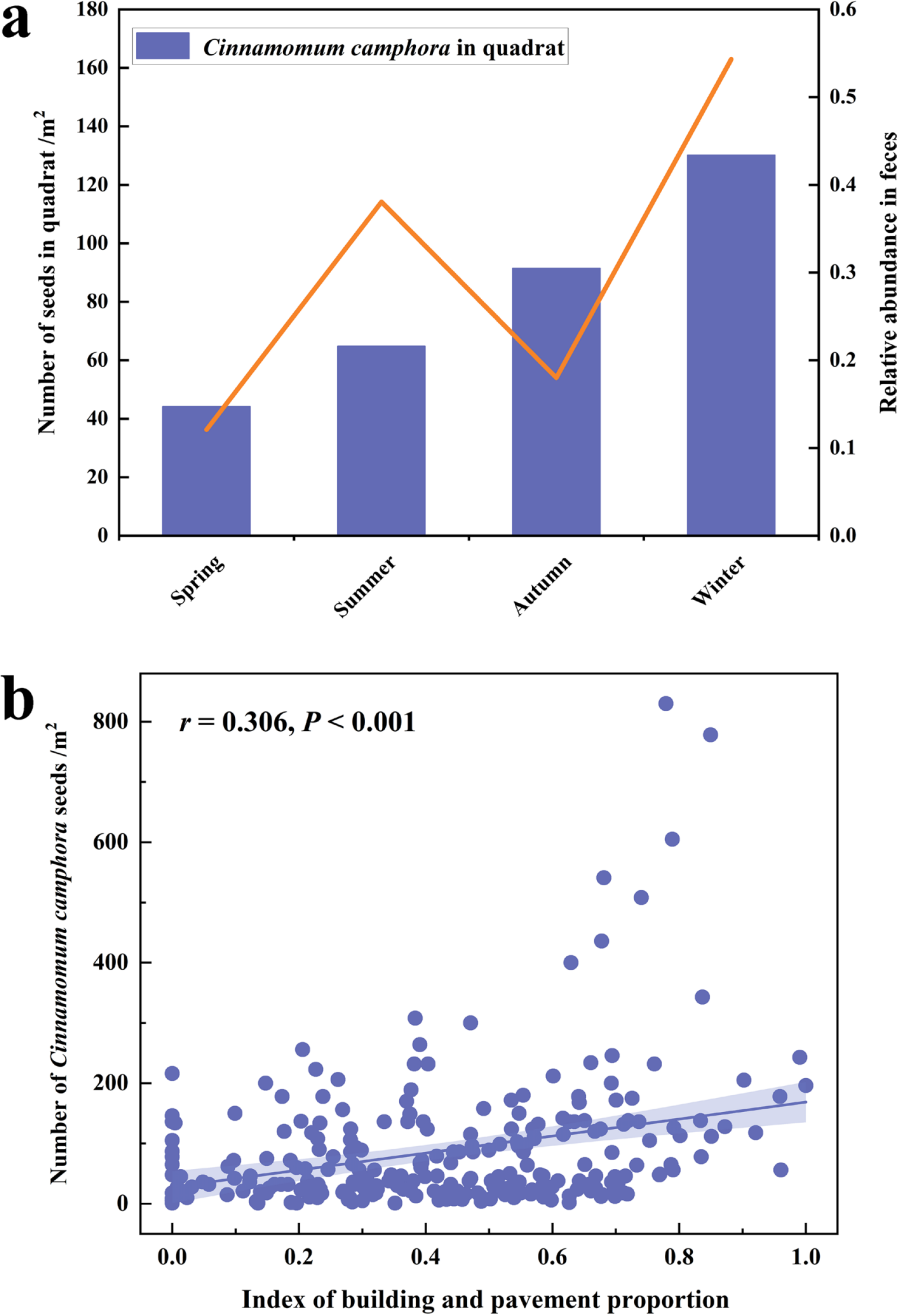
(a) The density of *Cinnamomum camphora* seeds in vegetation quadrats and the relative abundance in fecal samples. (b) The relationship between the number of *Cinnamomum camphora* seeds and the index of building and pavement proportion.

A total of 962 vegetation quadrats were obtained in the study area, including 173 vegetation quadrats in spring, 360 vegetation quadrats in summer, 205 vegetation quadrats in autumn, and 224 quadrats in winter. We detected a total of 81 species of plants assigned to 21 orders, 36 families, and 75 genera. The majority of plant species were obtained in spring quadrats, with a total of 62 plants surveyed. Only 43 plant species were observed in winter. We then retained only plants with relative coverage of more than 1% in each season for display (Figure S3). It should be noted that the dominant plant was *Zoysia japonica* for all seasons (relative coverage 42.23% in spring, 49.92% in summer, 50.15% in autumn, 57.52% in winter); *Zoysia japonica* was an artificial greening species in southern China. In addition, another greening species was *C. camphora* ; its seeds could be found everywhere and in large quantities (Figure 5a). Furthermore, there is a significant positive correlation between the number of *C. camphora* seeds and the index of building and pavement proportion (*r* = 0.306, *p* < 0.001) (Figure 5b). For other natural secondary plants, *Hypnum plumaeforme*, *H. sibthorpioides* , *V. philippica*, *Imperata cylindrica* , *Eleusine indica* , *Digitaria ciliaris* , and *P. distichum* can be found in all seasons. The dietary overlap is relatively high between different seasons based on Schoener's niche overlap indices (0.617–0.813) (Table S1).

**FIGURE 6:**
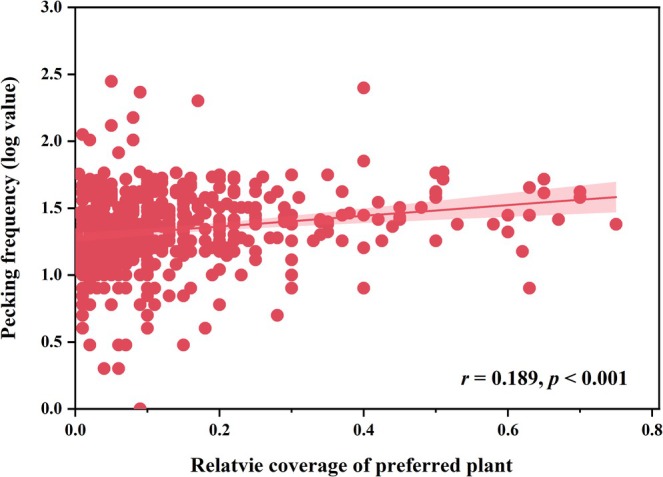
The relationship between the pecking frequency and the relative coverage of the 5 plants (the top 5 species of relative abundance in feces, excluding plants not present in vegetation quadrat) in quadrats.

**FIGURE 7:**
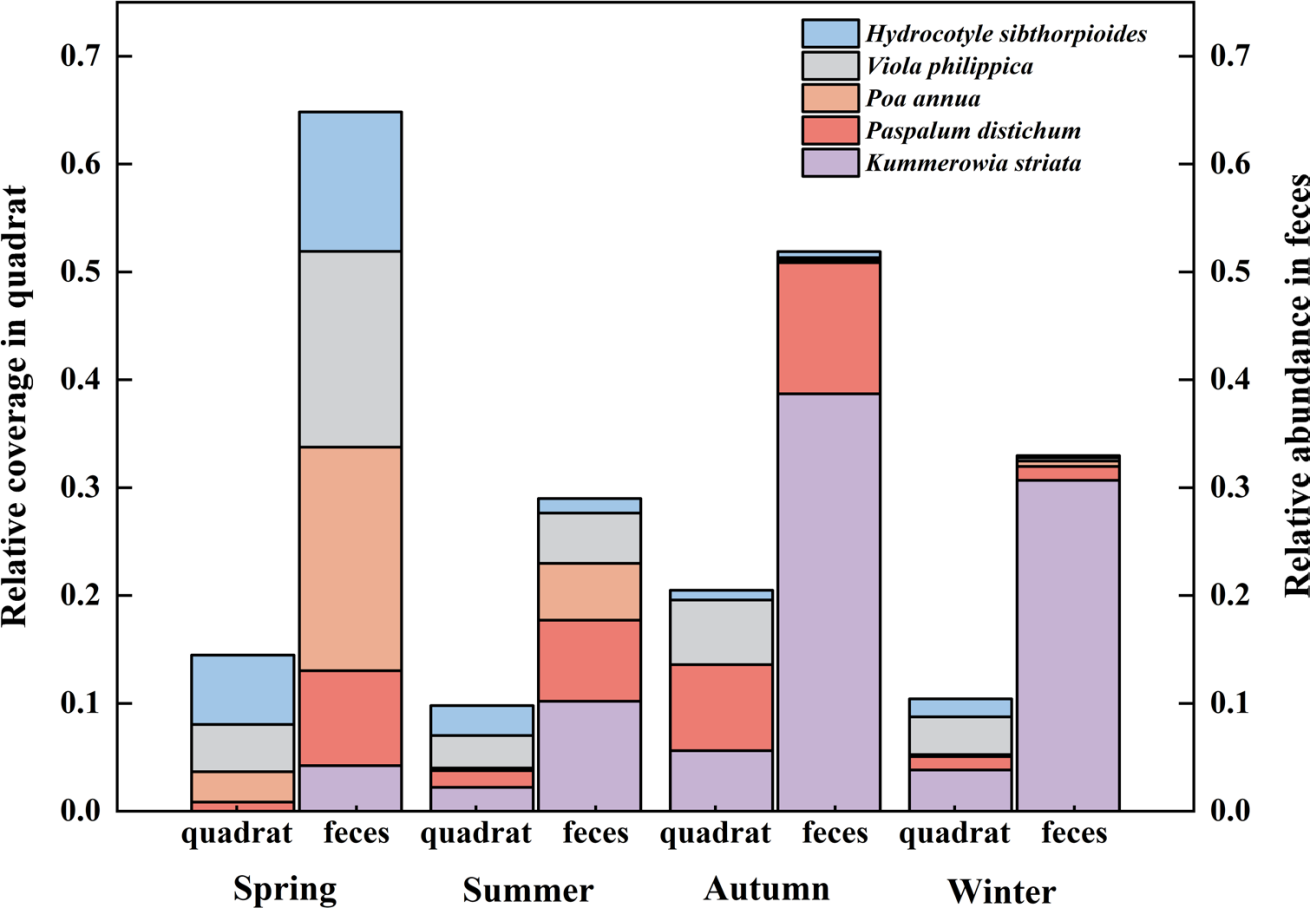
The variation of 5 plants (the top 5 species of relative abundance in feces, excluding plants not present in vegetation quadrat) in four seasons. The relative coverage in vegetation quadrat corresponds to the left *Y*‐axis, and relative abundance in fecal samples corresponds to the right *Y*‐axis.

However, the relative abundance of *Z. japonica* reached 5.10% in the spring feces, and it was hardly found in the rest of the seasons feces. We analyzed the relationship between the relative coverage of the five plants with the highest abundance of foraging throughout the year and the pecking frequency of the spotted doves in vegetation quadrats; the results indicated a significant correlation between the two variables (*r* = 0.189, *p* < 0.001; Figure 6). Furthermore, an analysis of seasonal change in the environment (quadrat) and diet (fecal) of the five most foraging plants showed that regardless of the low relative coverage of *P. annua* in quadrats, fecal samples had a lot of it in spring, as well as *P. distichum* , *V. philippica*, and *H. sibthorpioides* (Figure 7). In summer, as the quantity of Gramineae plants decreased, so did the relative abundance of feces, but it exhibited a high abundance of *C. camphora* seeds in feces (Figures 4a and 7). When the relative coverage of *K. striata* gradually rose in autumn and winter, it also presented a considerable proportion in fecal samples (Figure 7).

## 4 Discussion

### 4.1 Availability of Urban Resources Dictates the Diet

Urbanization is currently happening at a rapid pace, affecting the diet of herbivorous birds by altering the species composition of plant communities (Chan et al. 2020; Jarrett et al. 2020). Species that can quickly accept new foods may be better adapted to environmental variation when available resources change (Eccles et al. 2021). Spotted doves mainly foraged on farm crops (Gramineae) such as rice, wheat, and soybeans in rural areas (Pang 1980; Yan and Ma 1992). In this study, our results showed that urban spotted doves predominantly take greening plants as their main food (Table 2, Figure 5a). This is inconsistent with previous research. We found that spotted doves predominantly take *P. distichum* , *P. annua* , *V. philippica*, *H. sibthorpioides* , and *C. camphora* as their main food in spring; spring is also the season when spotted doves foraged the largest variety of species. In summer, the proportion of *P. annua* had decreased from 20.73% to 5.26%, and *V. philippica* also decreased from 18.15% to 4.66%; the same is observed with *H. sibthorpioides* (from 12.93% to 1.36%). But the proportion of *K. striata* gradually increased, from 4.22% in spring to 10.18% in summer, and up to 38.69% in autumn as well as 30.67% in winter. These greening plants have a greater ability to grow well in urban habitats. Among them, *C. camphora* seeds were the only plants taken in all seasons, because *C. camphora* was planted in quantities as street trees in Nanchang, China. Moreover, as the index of building and pavement proportion increased, so did the number of *C. camphora* seeds in the foraging quadrats (Figure 5b). This confirmed that even in highly urbanized areas, spotted doves engage in foraging. The diet of the urban spotted doves confirmed that they can take advantage of urban plants (including plants that were specifically planted). And the results supported our first hypothesis.

As urban boundaries expand, it is essential for fauna to exploit all available urban food sources to survive (Federspiel et al. 2017). In comparison to rural areas, there is a lack of crops in cities. But spotted doves focused on greening plants and had unique foraging resources in every season. And we found that spotted doves consumed breadcrumbs and rice leftovers occasionally. When ecosystems change, foraging habits of birds might also change. Furthermore, birds are able to assume new functional traits and roles in new environments. Similarly, in a study about Carolina chickadee ( *Poecile carolinensis* ), it has been found that urban chickadees were less fearful of new foods than rural chickadees and were able to accept new and novel foods in the city (Narango et al. 2017).

In contrast, the greening grass, *Z. japonica* , was not favored by spotted doves. Moreover, the index of Schoener's niche overlap between seasons was relatively high because of the absolute dominance of *Z. japonica* in greening plants (Figure S3; Table S1); it indicated that greening plants are still relatively singular in urban habitats. In addition, though the relative abundance of secondary plants like *P. annua* was low, it contributed a large amount of food quantity to spotted doves. All these suggested that there is a need to plant other grass species in urban greening, which can bring more food options for birds.

### 4.2 Dietary Selection Is Constrained by Seasonal Food Resources

Seasonal changes in food resources can lead to variations in dietary composition (Van Horne et al. 1998; Amponsah‐Mensah et al. 2019). In this study, it was most evident that spotted doves preferred to forage Gramineae plants in spring while preferring Leguminosae plants in autumn. The study of northern Idaho ground squirrel diet ( *Urocitellus brunneus* ) indicated that there is a difference between spring and summer because different plants are available at different times throughout the growing season, and squirrels may be forced to alter their diet based on plant phenology (Goldberg et al. 2020). Tang et al. (2022) found that Sichuan partridge (*Arborophila rufpectus*) can adjust their diet according to the availability of food resources and their own needs (among three periods of breeding, postbreeding wandering, and overwintering).

According to optimal foraging theory, when food is adequate and steady, individuals are more likely to be choosy and should select preferred food (Pyke et al. 1977). The urban habitats provide a more stable food resource for spotted doves, especially the most consumed *C. camphora* seeds, which are readily available almost year‐round but are not the first choice in all seasons (Figure 5a; Table 2), suggesting that spotted doves are selective about their foraging plants. When the coverage of the five plant species ( *K. striata* , *P. distichum* , *P. annua* , *V. philippica*, *H. sibthorpioides* ) in quadrats gradually increased, the pecking frequency of spotted doves also increased significantly (*p* < 0.001, Figure 6), implying that spotted doves have a preference for these five plant species. However, these 5 plants were not present in all seasons (Figure 7). When plants were increasing or decreasing in response to the phenological period, food preference would change according to the availability of food (Russo et al. 2020). In spring, there was an increase in the variety of plants available, and spotted doves foraged most on Gramineae ( *P. annua* , *P. distichum* ), Violaceae (*V. philippica*), and Araliaceae ( *H. sibthorpioides* ) (Table 2; Figures 4 and 7) because these plants are growing and tasseling from April to June (Soreng et al. 2022). In summer, many high‐protein Gramineae plants ( *P. annua* has the highest protein content (22.74%) of them and is also the plant taken the most in spring, *P. distichum* follewed it (16.37%), *Setaria viridis* has 11.04% protein cotent, *Imperata cylindrica* has 9.83% protein cotent; Wang et al. 2022) have declined in abundance, which leads to *C. camphora* seeds being a preferred choice for meeting breeding requirements. Summer is the time when *C. camphora* seeds are just ripening; the green seeds are more nutritious (18.15%, exceeds many Gramineae plants in study area), as confirmed by the study (Zhang and Xu 2001). Spotted doves focused on fruits of *K. striata* in autumn, which was the time of its increased coverage in quadrats (Table 2; Figure 7; McCurdy et al. 2015). These results confirm our second hypothesis. Our results suggested that dietary diversity was significantly lower in winter than in other seasons (Figure 3), indicating that winter food was scarce and constrained by resources. We found that spotted doves had the highest availability of *C. camphora* seeds in winter, while they were no longer the first choice in spring and autumn (Figures 2 and 4). Therefore, we believe that *C. camphora* seeds were a supplementary food resource in winter. In fact, the protein of most grasses decreases as the season progresses (Frase and Armitage 1989; Zhao et al. 2020); the availability of food resources during winter seems to be limiting for animals (Hagen et al. 2009; Sullins et al. 2018). Meeting high energy demands for warmth with the available food items of limited nutrients may be challenging (Sedinger 1997; Sullins et al. 2018; Fu et al. 2024). The *C. camphora* seeds are not only rich in protein but also extremely high in oil content (58.02%), making them a choice for both nutritional needs and defense against the cold weather (Li 2023). Therefore, *C. camphora* seeds become a supplementary food source for spotted doves, which can be consumed in large quantities when there is a shortage of their preferred plants to ensure survival and reproduction.

## 5 Conclusions

In summary, our results showed that the main food source of urban spotted doves was greening plants, which implicates that spotted doves can make use of resources in the urban environment. The dietary composition of spotted doves varied with increasing or decreasing plant resources. Only one common bird species was selected as the study subject, and the plant species that spotted doves foraged are limited, so it is possible that other birds cannot adapt to local greening plants. In fact, different herbivorous birds have unique foraging preferences; therefore, more ecological niches and plant species may be needed to satisfy the birds' needs, which requires further research. Despite our results confirming that spotted doves are able to utilize urban greening plants, the food preferences of spotted doves change according to the availability of plant resources. Plant growth conditions in different seasons need to be taken into account in order to rationalize the arrangement of greening plants in different phenological periods, avoiding the shortage of plant food in autumn and winter and providing sufficient resources for wildlife. Due to the relative homogeneity of plant species in urban environments and the high index of niche overlap between seasons, we still recommend increasing the diversity of greening plants in cities and tolerating weeds to some extent. This may give birds more foraging options and survival opportunities.

Finally, although our research showed that the diet of the spotted doves is well suited to the artificial greening of Nanchang City, this does not mean it would be applicable in other cities (i.e., high‐latitude or high‐altitude city). Because each city has different vegetation based on climate and culture, we still need to focus on the food resource of birds in other urban environments. It is important to focus on the life history characteristics of local urban dominant plant species, to provide appropriate suitable habitats and food for wildlife, thus providing strong support for urban wildlife conservation and sustainable survival.

## Author Contributions

**Mengjie Lu:** data curation (equal), methodology (equal), visualization (equal), writing – original draft (equal). **Xueli Zhang:** data curation (equal), investigation (equal), writing – original draft (equal). **Jiahan Ruan:** data curation (equal), investigation (equal), software (equal). **Wanyou Li:** data curation (equal), investigation (equal), software (equal). **Mingwang Chen:** data curation (equal), investigation (equal). **Long Ma:** data curation (equal), investigation (equal). **Zhen Zhang:** data curation (equal), investigation (equal). **Bicai Guan:** software (equal), visualization (equal). **Luzhang Ruan:** conceptualization (equal), funding acquisition (lead), writing – review and editing (equal).

## Conflicts of Interest

The authors declare no conflicts of interest.

## Supporting information


Data S1

## Data Availability

The data that support the findings of this study are available in Figshare at https://doi.org/10.6084/m9.figshare.28616126.
