# Supplementary material for: Selection and Adaptation to Urban Food Resources Promote Spotted Dove (Streptopelia chinensis) as a Common Species in Urban Habitats
Source: Ecol Evol. 2025 Jul 10;15(7):e71773. doi: 10.1002/ece3.71773 (PMC12242857; doi:10.1002/ece3.71773)
Supplement: Supplementary file 1 — Data S1 [file ECE3-15-e71773-s001.docx]

**Supplementary Materials**

**Figure S1** Rarefaction curve for four season samples of Spotted doves in four seasons.


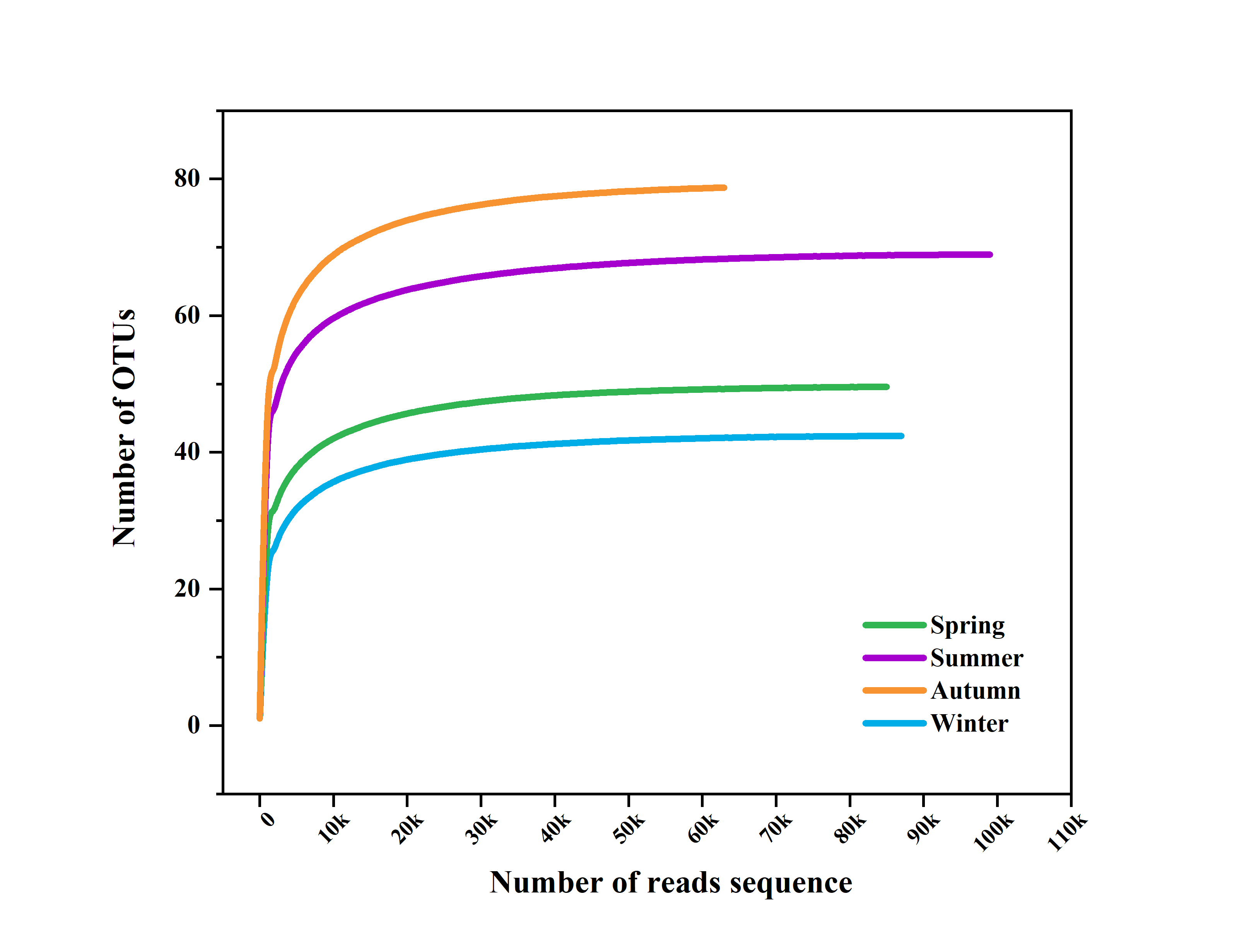


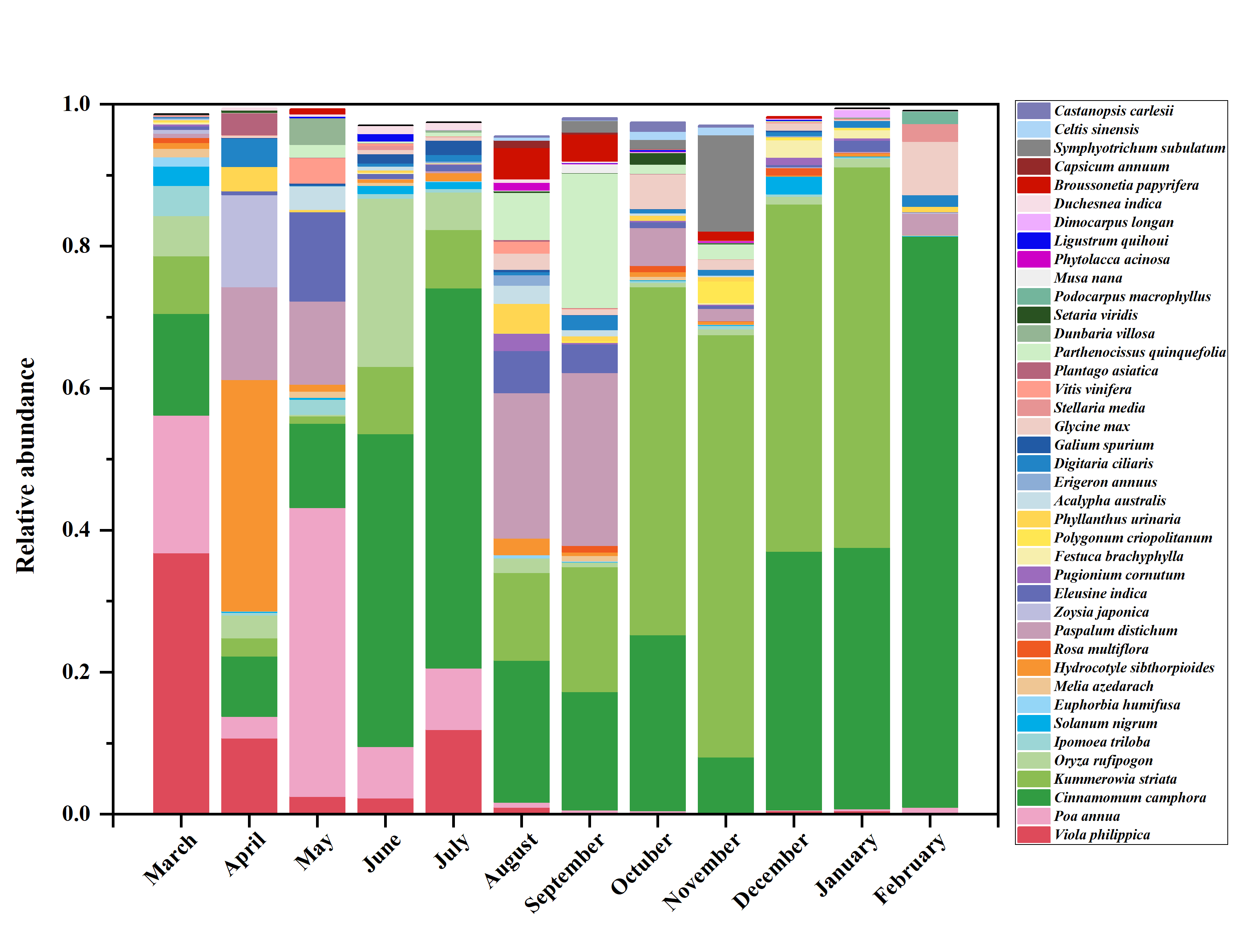
**Figure S2** Barplot showing the plant items (relative abundance > 1% ) in different months.

**Figure S3** Relative coverage of common plants in vegetation quadrats (relative coverage > 1%). The outermost ring indicates winter plants and the innermost ring indicates spring plants.


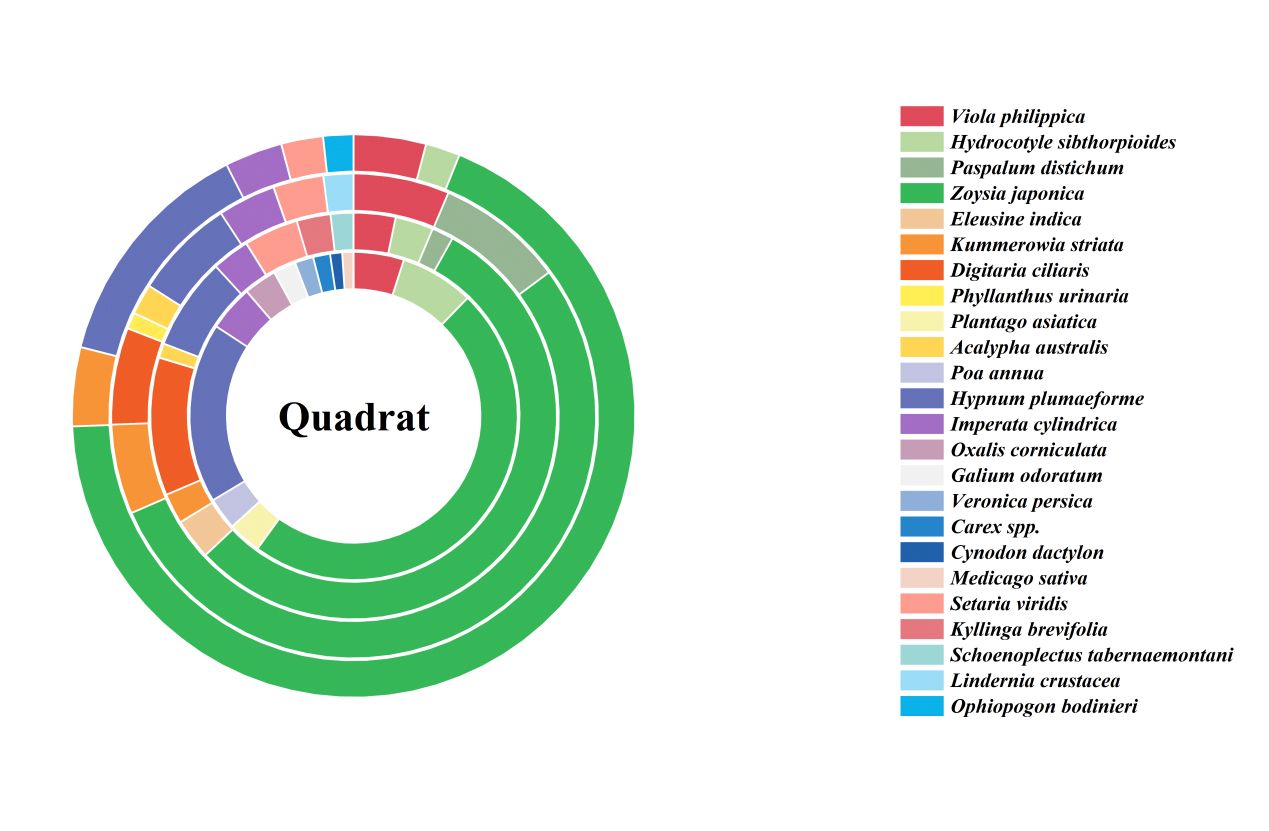


**Table S1** Schoener's niche overlap indices in quadrats. Overlap values closer to 1.0 indicate more similar diets and overlap values closer to 0.0 indicate less overlap.

|  | Spring | Summer | Autumn | Winter |
| --- | --- | --- | --- | --- |
| Spring | * | * | * | * |
| Summer | 0.651 | * | * | * |
| Autumn | 0.617 | 0.813 | * | * |
| Winter | 0.708 | 0.748 | 0.742 | * |
